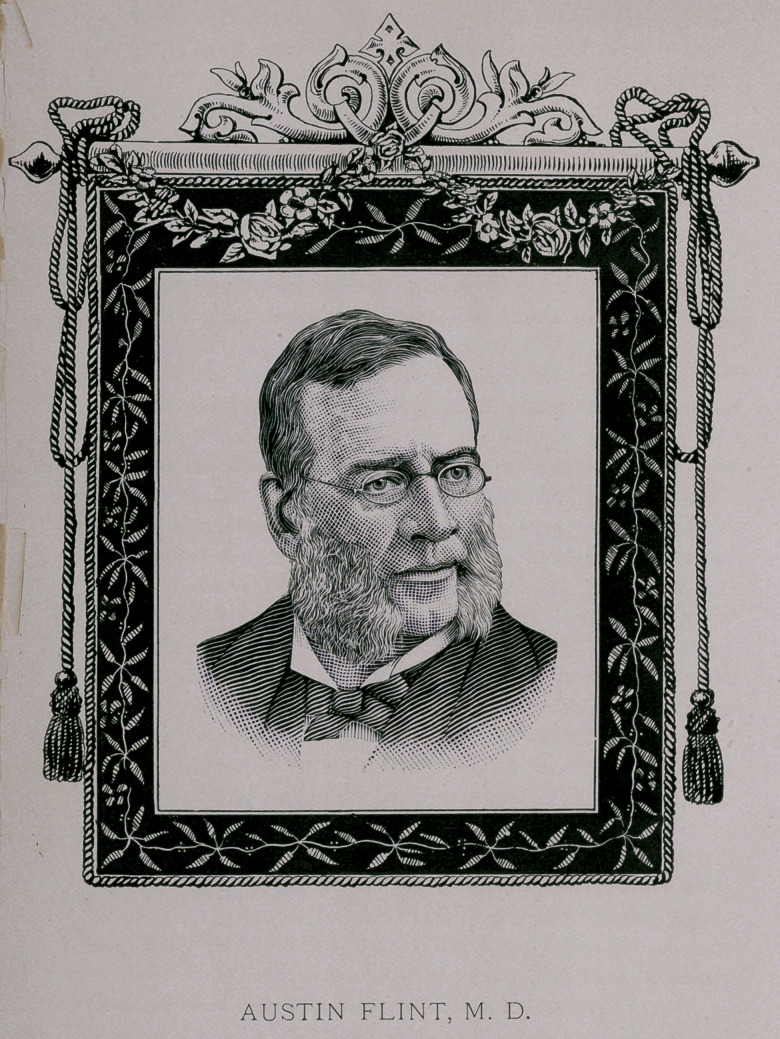# Austin Flint, M. D., LL.D.

**Published:** 1886-04

**Authors:** 


AUSTIN FLINT, M. D.

### Editorial.

### Austin Flint, M. D., LL. D.

This country, and in fact the whole world, will mourn the loss of this distinguished physician, who died at his home in New York, March 13th, at the age of 74. It can be truthfully said of Dr. Flint, that he was the most celebrated and truest type of the American physician. In his methods of diagnosis and manner of dealing with disease he had no equal. It will indeed be difficult to find among so many eminent men one who can successfully fill his place. Few men possess the scientific knowledge, and fewer, if any, possess this knowledge and the ability to apply it. He was a man of such noble bearing that his presence alone inspired confidence. He has made no great discoveries which could make him famous: but his earlier, and, in fact his later writings contained many scientific truths never before so forcibly presented to the profession. As a leader he had no equal. He had an indomitable will, and yet would listen to reason. "He had that fortunate combination of elements in his character, which enabled him to achieve the completest success attainable, without awakening envy or creating enemies. Few men have been so justly eminent, and yet so widely beloved and admired."

Dr. Flint's ancestors, for many generations, were physicians of considerable eminence, and he early learned the qualities necessary to be successful as a medical man. His opportunities for education were excellent, and so, early in life, he took a foremost rank in the profession. He founded the Buffalo Medical College, and, in 1845, the Buffalo Medical Journal. He edited this for ten years, and "his contributions to this periodical attracted attention on account of their unusual worth."

Here it is he achieved his first success. While resident here, he labored part of the time in Chicago, Louisville and New

Orleans, and each come in for a share of honor in his great professional attainments. His intense devotion to what he deemed the best interests of the profession throughout the country, is explained by his residence in so many places and sections of the country. This explains, too, the wonderful clearness with which he discussed, in his writings, the diseases peculiar to certain localities. In Europe, as well as in America, he was regarded as the most celebrated of all American physicians.

As Editor of this Journal, the first editorial which Dr. Flint wrote was forcible, and true to-day as it was forty years ago. We reproduce it here, thinking it may prove of interest:

INTRODUCTORY.

On the occasion of introducing to the medical public the first number of a new journal, it will be expected of the editor to give some reasons for its appearance, and to premise some account of the objects to which it will be devoted.

With regard to the first, we have only to offer the conclusion, which has been formed, after due reflection, that a medical journal, if it commend itself to the approval and interest of those to whom it will address itself for encouragement and support, may be satisfactorily sustained, and, in various points of view, become subservient to useful ends. We might be somewhat distrustful of this conclusion, if it were peculiar to ourselves; but it is also entertained by several professional brethren, who have felt sufficient confidence in its correctness, to unite in originating this undertaking, and pledge themselves to secure a fair trial of its practicability.

It will readily be acknowledged that for the more voluminous and elaborate journals, in medical as in other departments of knowledge, we must look to the larger cities, where the elements and facilities for their preparation and diffusion are to be found in the greatest abundance. But without any derogation from the superior claims of these, there are many reasons why they do not and cannot accomplish all the objects to be derived from periodical literature. Of these reasons, we may hereafter take occasion to speak more particularly, and to discuss their respective merits. But assuming, for the present, their existence and validity, it has seemed to ourselves and others that Buffalo is, in many respects, a desirable location for a medical journal. This opinion is based on its present and prospective size and resources: its relations with the east and west, through canals and railroads on the one hand, and the chain of the great Lakes, with their numerous tributaries, on the other. It is believed that sufficient material to commence an enterprise of this kind may be derived from sources which will be constantly increasing and improving, and that a medical journal may do much, not only toward making available the material which already exists, but to render its future availability and improvement commensurate with its increase. By means of facilities for rapid and extensive communications on every side, our location is peculiarly favorable for the collection and diffusion of facts from a wide circuit, and the interchange of views and opinions among members of the profession, not only in this section of country, but situated at points remotely distant from each other.

We have also, in this project, ventured to act upon the belief (and we hope it may be expressed without undue presumption) that the medical fraternity, whose interest and assistance will be especially solicited, are not deficient in the ability, or the disposition to contribute their proportion of effort for the advancement of the legitimate ends and interests of our common science.

With these views, the JOURNAL is commenced. As before intimated, its continuance is secured for a sufficient period, to provide a practical test of its feasibility and utility. The result of the experiment will determine whether it is to be abandoned as a premature or misplaced project; or, on the other hand, whether it is to be continued on a larger scale.

In conducting the JOURNAL, we shall, in the first place, devote as much space as practicable to original communications. Cases are constantly occurring, under the observation of practical men, which are interesting from their novelty, and which contribute either to develop or confirm important truth. We shall be glad to receive reports of such cases, and communicate them for the benefit of the whole profession. A great number of useful thoughts and ideas originate in the minds of observing and reflecting practitioners, which it is desirable to throw into the crucible of medical literature, there to produce results according to their intrinsic value. We offer our JOURNAL as a medium of communication, in this respect, between the minds of individuals and the common mind of the profession. Of particular points of medical science, upon which we may especially desire to collect information, we shall take occasion to speak, from time to time, as they may suggest themselves. But, in general terms, the first object will be to gather up from different sources important matter, much of which would otherwise be lost, adding it to the common stock of knowledge, and thereby contributing to the progress of medical science.

In the second place, we shall endeavor to supply to those who may become our patrons, condensed intelligence of discoveries, improvements and new views in medicine, surgery and collateral branches of science, which may be communicated to the profession in this country or abroad through periodicals and books. We shall have access to several of the most prominent journals, domestic and foreign, and shall avail ourselves of their contents for the benefit of our readers. In doing this, we shall not undertake to conflict with their individual claims to the esteem and patronage of the profession; still less, in any measure, to supersede the importance of resorting to them more directly as sources of valuable information. So far from this, we shall strive to promote their circulation, by endeavoring to diffuse a more general appreciation of their value.

Our pages will be open to interchange of opinions and free discussion, not only of subjects strictly belonging to medical science, but of any involved in the progress or interests of the medical profession. Medical Education, Medical Legislation, Quackery,

Medical Ethics, and the varicus plans of Medical Reform, are among the subjects which, at the present time, furnish questions of interest and moment, both as regards the profession and the public. We shall be desirous of appropriating to articles upon these topics as much space as our limits will allow, and to afford an opportunity for the expression of different views and opinions, without restraint, excepting by the rules of decorum and propriety.

We would add that the JOURNAL is pledged to no interests apart from those which relate exclusively to the progress of medical science and the advancement of the medical profession. It is not instituted for any sectional objects or partisan views; but to serve as an organ for the impartial and untrammelled utterance of opinion on any matters pertaining, directly or indirectly, to its professed objects.

In conclusion, the editor desires to say, in his own behalf, that he looks for the means of success in this undertaking, not to his own exertions so much as to the assistance which he expects to receive from others. He enters upon the duty, however, with a determination to do what he can personally to contribute to its success, and is stimulated with the hope that this will be such as to authorize, ere long, the enlargement of the JOURNAL, so that he may be able to render it much more acceptable and useful than is compatible with its present restricted limits.

BUFFALO, June 1, 1845.

In conclusion, we may be permitted to say that the high standard of editorial duty outlined by Doctor Flint has been consistently maintained by successive editors during the last forty years; and, under its present management, the principle has been firmly upheld that—"*It is not instituted for sectional objects or partisan views; but to serve as an organ for the impartial and untrammelled utterance of opinion on any matter pertaining, directly or indirectly, to its professed objects.*"

## Figures and Tables

**Figure f1:**